# PDK4 Constitutes a Novel Prognostic Biomarker and Therapeutic Target in Gastric Cancer

**DOI:** 10.3390/diagnostics12051101

**Published:** 2022-04-27

**Authors:** Zimu Zhang, Shiyuan Han, Siwen Ouyang, Ziyang Zeng, Zhen Liu, Juan Sun, Weiming Kang

**Affiliations:** 1 Department of General Surgery, Peking Union Medical College Hospital, Chinese Academy of Medical Sciences & Peking Union Medical College, Beijing 100730, China; zhangzm920728@163.com (Z.Z.); xuexqpumc@sina.com (S.O.); zengzypumch@163.com (Z.Z.); chengepumch@sina.com (Z.L.); sunjuanpumch@163.com (J.S.); 2 Department of Neurosurgery, Peking Union Medical College Hospital, Chinese Academy of Medical Sciences & Peking Union Medical College, Beijing 100730, China; edisonted1226@sina.com

**Keywords:** gastric cancer, PDK family, biomarker, cancer metabolism, prognostic model, tumor growth, invasion and migration

## Abstract

Gastric cancer (GC) is one of the most prevalent and deadly malignancies worldwide. We aimed to assess the functional role and clinical significance of pyruvate dehydrogenase kinase (PDK) in GC and explored the underlying mechanisms. The bioinformatics method was used to investigate the expression of PDKs in GC, the effect on clinical outcomes, enriched pathways, interactive network, and the correlation between PDK4 and immune infiltration. Next, PDK expression in the GC cells and tissues were verified by qRT-PCR and western blotting. A Cell Counting Kit-8 (CCK8), colony-formation, Flow cytometry, Transwell and wound healing assays were carried out to evaluate the influence of PDK4 on cell proliferation, invasion and migration. Among PDKs, PDK4 expression was aberrant in GC and identified as an independent prognostic factor. GO analysis, GSEA, and PPI showed that PDK4 expression may regulate cell adhesion, metal ion transport, synaptic activity, and cancer cell metabolism in GC. Analyses of immune infiltration showed that PDK4 correlated with the abundant expression of various immunocytes. Finally, we verified that upregulation of PDK4 expression enhanced the ability of GC cells to proliferate, migrate, and invade. In conclusion, PDK4 was identified as a potential candidate diagnostic biomarker and therapeutic target for GC patients.

## 1. Introduction

Despite its decline in the incidence and mortality over the past five decades, gastric cancer (GC) remains one of the most prevalent and deadly malignancies worldwide, with over one million new cases (5.6%) and 769,000 deaths (7.7%) in 2020 [1]; GC ranked fifth in incidence and fourth in mortality among all malignant cancers [2]. In China, following lung cancer, GC is the second most common cancer diagnosis (67.9/100,000), which constitutes 60% of all GC cases worldwide, and GC is the third leading cause of cancer-related deaths (48.9/100,000) [3]. Although progress in the treatment of GC has been made, including surgical excision and adjuvant chemoradiotherapy, the long-term prognosis of GC patients remains unfavorable due to the high level of malignancy and likelihood of metastasis [4]. Thus, the development of novel therapeutic strategies necessitates the elucidation of the underlying molecular mechanisms that regulate GC development and progression.

Altered energy metabolism is a biochemical fingerprint of cancer cells and represents one of the “hallmarks of cancer” [5]. Malignancies undergo metabolic reprogramming to utilize glycolysis even in the presence of abundant oxygen to meet the anabolic requirements of the rapid growth of cancer cells, which is called the “Warburg effect” [6]. Furthermore, glycolysis increases lactic acid production, thereby creating an acidic microenvironment, wherein the extracellular matrix is extremely unstable and tumor cell metastasis will be boosted [7]. Thus, heavy reliance on glycolysis could constitute a feasible target for amelioration and for unearthing novel anticancer strategies [8]. The pyruvate dehydrogenase kinase (PDK) family inhibits the entry of pyruvate into the tricarboxylic acid (TCA) cycle by impairing the activity of the key executor pyruvate dehydrogenase (PDH), and thereby switches energy derivation to cytoplasmic glycolysis in lieu of mitochondrial oxidation [9]. Four PDK isoenzymes (PDK1, 2, 3, and 4), which possibly serve as antitumor targets because of their significance in energy production, have been identified in mammalian specific tissues [10]. Disturbed PDK expression, which contributes to tumor cell proliferation, migration, and invasion, has been previously reported in multiple cancers, including colon, bladder, breast, ovarian cancers, etc. [11,12,13,14]. However, the underlying mechanistic details of how the PDK family interacts with GC development and progression have not been investigated, as the Warburg effect constitutes a vital player in GC metabolism.

In this study, we used bioinformatics to comprehensively investigate the prognostic value and therapeutic potential of the PDK family (especially PDK4) in GC patients. Furthermore, we tried to ascertain the possible mechanisms underlying PDK4 function in GC and to illustrate the significance of PDK4 in GC tumorigenesis and progression. Moreover, we used quantitative real-time transcription-polymerase chain reaction and western blotting (WB) to verify PDK4 expression in human GC cell lines, the immortalized gastric mucosal epithelial cell line (GES-1), and in human samples. We upregulated and downregulated PDK4 expression in GC cells and then validated the impacts on GC cell proliferation, invasion, and migration by using the Cell Counting Kit-8, colony formation, flow cytometry, Transwell, and wound healing assays.

## 2. Materials and Methods

### 2.1. Public Data Acquisition and Preprocessing

We collected RNA-seq profiles and clinical data from the official website of The Cancer Genome Atlas (TCGA), and included 375 tumor samples and 32 normal tissues. Moreover, we incorporated normal samples from the Genotype-Tissue Expression Project (GTEx). The downloaded gene expression data were transformed into the transcripts per million reads (TPM) format for subsequent analyses.

### 2.2. Analysis of PDK Mutations and Prognosis

The cBioPortal (https://www.cbioportal.org (accessed on 8 September 2021)) website can be used to explore, analyze, and visualize cancer genome data. The genetic alteration data of the PDK family was obtained from the cBioPortal with a z-score ±2.0, and their association with survival were analyzed to identify their prognostic value (Stomach Adenocarcinoma, TCGA, Nature 2014).

### 2.3. Survival Analysis

The correlation of PDK expression with overall survival (OS) and disease-specific survival (DSS) of GC patients were evaluated using the survival R package (v.3.2-10) and the survival curves were generated by the survminer R package (v.0.4.9).

### 2.4. Multivariate Cox Regression Analysis

To determine the effects of PDK expression on GC survival, we performed multivariate Cox proportional hazard regression analysis to assess independent prognostic factors of OS using the survival R package (v.3.2-10). The forest plot via ggplot2 R package (v.3.3.3) was applied to visualize the results.

### 2.5. Construction and Evaluation of the Nomogram

A nomogram was established based on the optimal multivariate Cox regression analysis to predict the 1-, 3-, and 5-year OS probabilities of GC patients via the rms R package (v.6.2-0) and survival R package (v.3.2-10). The relationship between the predicted and observed risks for the outcomes of the nomogram was graphically displayed via calibration plots, and the 45° line represented the optimal predictive values.

### 2.6. Analysis of DEGs between the PDK4-High- and -Low-Expression Groups

Patients identified from the TCGA dataset were assigned to the PDK4 low- and high-expression groups. Differentially expressed genes (DEGs) between the two groups were identified with the DESeq2 R package (v.1.26.0). Genes with an adjusted *p*-value < 0.05 and absolute fold change >1.5 were considered as statistically significant. All DEGs were presented in volcano plots via ggplot2 (v.3.3.3).

### 2.7. Enrichment Analysis

To determine the biological characteristics of transcriptomic data, Gene Ontology (GO) analyses, including biological process (BP), cellular component (CC), and molecular function (MF) analyses, were performed using the clusterProfiler R package (v.3.14.3); *p* < 0.05 represented statistically significant differences.

Gene set enrichment analysis (GSEA) is a computational method based on the entire gene expression matrix that determines whether a priori defined set of genes shows statistically significant and concordant differences between two biological states. Statistical analysis and graphical plotting were performed using the clusterProfiler (version 3.14.3). The Molecular Signatures Database (MSigDB) Collection (c2.cp.v7.2.symbols.gmt) was selected as the reference gene set. The threshold value was set as an adjusted *p* < 0.05 and false discovery rate (FDR) < 0.25. We utilized the normalized enrichment score (NES) to rank the enriched pathways.

### 2.8. Protein–Protein Interaction (PPI) Network Construction and Hub Gene Identification

To construct the PPI network, the DEGs were imported into The Search Tool for the Retrieval of Interacting Genes/Proteins (STRING, https://string-db.org/ (accessed on 17 October 2021)) database and a confidence score of >0.4 was set as the threshold. Cytoscape (v3.7.1) was used to visualize the PPI network and to analyze the significant modules and hub genes. The Mcode application was used for checking the clustering modules (degree cutoff = 2, node score cutoff = 0.2, maximum depth = 100, and k-score = 2), whereas the CytoHubba plug-in was used to screen the PPI network and genes with a degree >10 and these were identified as hub genes.

### 2.9. Immune Infiltration Analysis

Using the GSVA R package (v.1.34.0), the single-sample Gene Set Enrichment Analysis (ssGSEA) was applied to calculate the association between PDK4 and the levels of 24 types of immune cells between the PDK4-high- and -low-expression groups as well as to compare the different expression of these cells. Moreover, the immune, stromal, and ESTIMATE scores were calculated and compared by estimate R package (v.1.0.13).

### 2.10. Cell Culture

The human GC cell lines (MKN45, HGC27, NCI-N87, and AGS) and the immortalized gastric mucosal epithelial cell line GES-1 were obtained from the Cell Center of the Chinese Academy of Medical Sciences (Peking, China). These cells were cultured at the Roswell Park Memorial Institute (RPMI) in 1640 high-glucose medium supplemented with 10% fetal bovine serum (FBS) and 1% penicillin–streptomycin (Gibco, Carlsbad, CA, USA) under 37 °C in a humidified incubator with 5% CO_2_.

### 2.11. Gastric Tissue Specimens

In total, nine pairs of fresh cancer samples and matched adjacent non-tumorous gastric mucosa tissues were collected from patients who underwent radical resection at the Peking Union Medical College Hospital (PUMCH) from September 2020 to April 2021. No patient preoperatively received neoadjuvant chemotherapy or radiotherapy. All samples were frozen in liquid nitrogen within 30 min of removal, and stored at −80 °C until use.

### 2.12. RNA Extraction and Quantitative Real-Time Polymerase Chain Reaction (qRT-PCR)

Total RNA from tissues and cell lines was extracted using TRIzol Reagent (ThermoFisher, Waltham, MA, USA) and reverse-transcribed into cDNA by using a cDNA synthesis kit (Takara, Tokyo, Japan). Real-time RT-PCR was performed using SYBR Premix Ex Taq II (Takara, Tokyo, Japan). PCR amplification was carried out utilizing the ABI 7500 real-time system, under the following conditions: 95 °C for 10 min, followed by 40 cycles of 95 °C for 15 s, and 60 °C for 1 min. The 2-ΔΔCt method was used to quantify relative mRNA expression levels in GC cells and in the control, with β-actin as the endogenous reference. The RNA primers were as follows: PDK4 forward, 5′-CAATGGCACAAGGAATCATAGA-3′; PDK4 reverse, 5′- TCATCAGCATCCGAGTAGAAAT-3′; β-actin forward, 5′-GCATCGTCACCAACTGGGAC-3′; β-actin reverse, 5′-ACCTGGCCGTCAGGC AGCTC-3′.

### 2.13. Protein Extraction and Western Blot Analysis

Cells were lysed in RIPA buffer (Beyotime, Shanghai, China) with 1% phenylmethylsulfonyl and then denatured. Protein concentrations were quantified by BCA assay. The lysate was separated via 10% sodium dodecyl sulfate polyacrylamide gel electrophoresis (SDS-PAGE), and the proteins obtained were transferred to polyvinylidene difluoride (PVDF) membranes which were blocked with tris-buffered saline and 0.1% Tween 20 (TBST) containing 5% non-fat milk for 1 h at 37 °C and subsequently incubated with the following primary antibodies at 4 °C overnight: mouse anti-PDK4 monoclonal antibody (1:1000; ab110336, Abcam); mouse anti-GAPDH (1:1000; YM3029, Immunoway). The next day, the PVDF membranes were washed three times with TBST buffer and incubated with horseradish peroxidase (HRP)-conjugated anti-mouse secondary antibody (1:3000; SA00001-1, Proteintech) for 1 h at room temperature. Signals were visualized using an enhanced chemiluminescence reagent (ThermoFisher, Waltham, MA, USA) according to the manufacturer’s instructions. The relative protein expression levels were normalized with GAPDH as the standard reference.

### 2.14. Lentivirus Construction and Infection

The GV230 vector and GV248 vector were used to construct a recombinant PDK4 overexpression lentivirus and a knockdown lentivirus, respectively. HGC27 was infected with the knockdown lentivirus and the AGS cell was infected with the overexpression lentivirus according to the manufacturer’s protocol. Stable cells lines were selected using puromycin (Gibco, Carlsbad, CA, USA) with a concentration of 5 μg/mL. The efficiency was verified by WB.

### 2.15. Cell Proliferation Assay

The cell proliferation ability was assessed using the Cell Counting Kit-8 (CCK-8; Beyotime, Shanghai, China). The indicated number of GC cells was plated in 96-well plates. The 0-h time point was measured at the time of transfection. Other viable cells grown in 96-well plates were monitored after being cultured for 24, 48, 72, and 96 h. CCK-8 solution (10 μL/well) was added to each well 1 h before the assay and the plates were incubated for 2 h. The optical density value of 450 nm was detected on a microplate reader (Bio-Tek, Winooski, VT, USA).

### 2.16. Colony-Formation Assay

Stably transfected GC cells were seeded in each well of a 6-well plate in triplicate and incubated for 14 days. The medium was changed at three-day intervals. At the end of the incubation period, the cultures were washed, fixed with 4% paraformaldehyde for 30 min, and stained with crystal violet for 15 min. Visible colonies were counted, photographed, and subsequently analyzed.

### 2.17. Flow Cytometric Detection of Cell-Cycle Distribution

GC cells that underwent different treatments were harvested and then fixed with 75% ethanol at 4 °C overnight. Before detection, we rinsed the cells and stained them with 1 mg/mL propidium iodide (PI) solution (Biosciences, Beijing, China) containing RNase A for 0.5 h at 37 °C. Next, the distribution of cell-cycle phases was determined using the NovoCyte Flow Cytometer (ACEA Biosciences, Beijing, China). Thereafter, the percentages of cells in the G0/G1, S, and G2/M phases were analyzed with NovoExpress (v.1.1.0).

### 2.18. Transwell Assay

The invasive and migratory abilities of GC cells were detected using 24-well Transwell cell culture chambers (8.0-μm pore size, Costar, Cambridge, MA, USA). Differentially treated cells in 200 μL serum-free medium were added into the upper chambers at an appropriate density. Next, 600 μL medium containing 20% FBS was added into the lower chambers. For the cell invasion assay, the insert membranes were pre-coated with Matrigel (BD Biosciences, San Diego, CA, USA). After 24-h incubation, the non-invading or non-migrating cells on the upper membrane surface were gently removed with cotton balls, and the cells that passed through the membrane were fixed with 4% paraformaldehyde and stained with crystal violet for 15 min. The numbers of invaded cells were counted in five randomly selected fields (magnification, ×10) under a microscope.

### 2.19. Wound Healing Assay

The migration ability of GC cells was assessed using the wound healing assay. GC cells with different transfections were plated at a density of 3 × 105 cells/well and grown in 6-well plates in complete medium. When the cells reached 70–90% confluence, cell monolayers were scraped with a sterile pipette tip (200 μL) to generate three parallel, linear wounds. After an additional 24 and 48 h of culture, representative scratched areas were photographed, and the sizes of the wounds were measured and analyzed.

### 2.20. Statistical Analysis

The Wilcoxon rank-sum test was used to compare the expression of PDKs between the tumor and normal samples. Experimental data were performed in triplicate and represented as mean ± standard deviation. Two sets of independent samples were compared using the Student’s *t*-test. The ANOVA was used to compare the differences among the three groups. The Kaplan–Meier method was used to construct survival curves, and the log-rank test was used to analyze the differences between survival curves. We used Spearman’s correlation analysis to evaluate the relationship between PDK4 expression and tumor-infiltrating immune cells. A two-tailed *p*-value less than 0.05 indicated statistical significance for all analyses. All bioinformatical analyses and visualization were performed using R (version 3.6.3). GraphPad Prism (version 8.0.0) was used to perform experimental data analysis.

## 3. Results

### 3.1. Aberrant PDK Expression in Various Cancers

**Figure 1:**
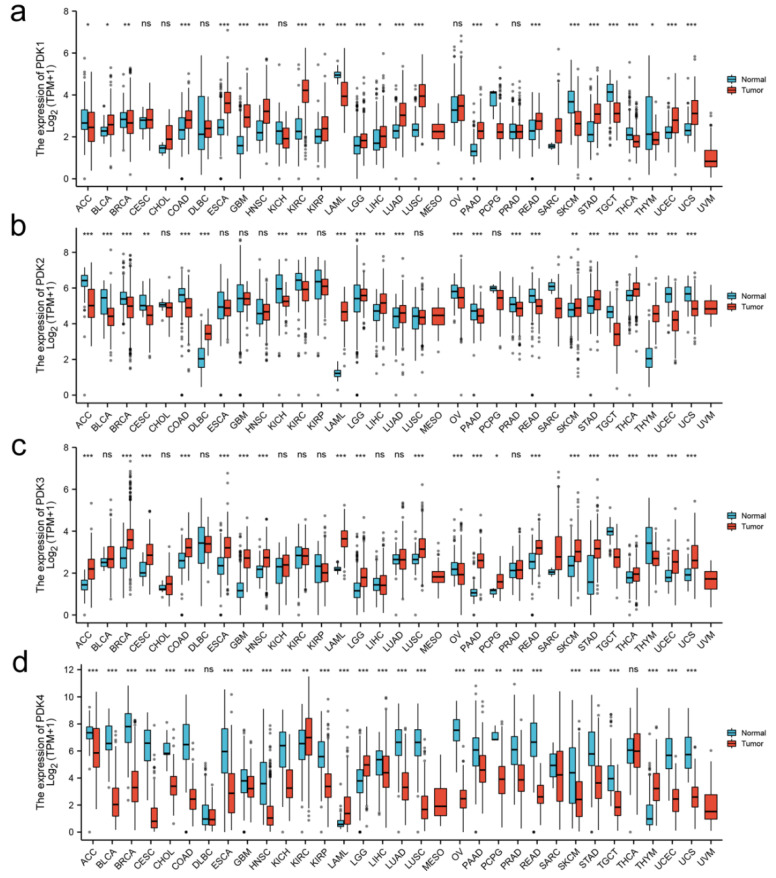
Expression levels of the PDK family in cancers. The figure (**a**–**d**) showing upregulated (red) or downregulated (blue) mRNA expression of PDK family of different cancers compared with the corresponding normal tissues in the TCGA and GTEx database. * *p* < 0.05, ** *p* < 0.01, *** *p* < 0.001. PDK4, pyruvate dehydrogenase kinase; TCGA, The Cancer Genome Atlas; GTEx, The Genotype-Tissue Expression Project.

According to the TCGA and GTEx database, the pan-cancer analyses showed that the PDK expression levels of several cancers were aberrant when compared with the expression levels in normal tissues (Figure 1), including GC (stomach adenocarcinoma (STAD)). Next, when compared with normal tissues, we found that the expressions of PDK1/2/3were significantly upregulated in GC samples whereas PDK4 expression was significantly downregulated (*p* < 0.001 for all).

### 3.2. Analysis of PDK Genetic Alteration and Association with Prognosis in GC

**Figure 2:**
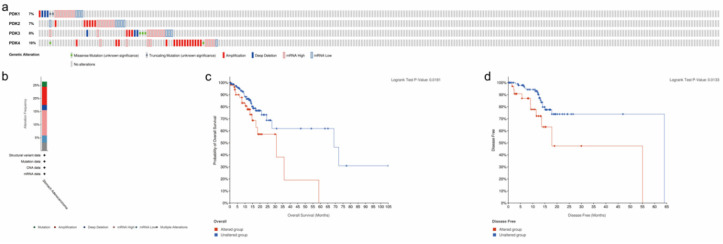
Analysis of PDK mutation and its association with prognosis. Summary plot (**a**,**b**) displaying alterations of PDK family in 258 GC patients. Patient survival status showing GC patients with altered PDK associated with poor OS (**c**) and DFS (**d**). GC, gastric cancer; OS, overall survival; DFS, disease-free survival.

We explored the genetic characteristics of differentially expressed PDKs using cBioPortal and found that PDK1, PDK2, PDK3, and PDK4 expression were altered in 7%, 7%, 8%, and 10% of the queried GC samples, respectively (Figure 2a). Moreover, genetic alterations in the PDK family were significantly associated with unfavorable OS and DFS in GC patients (*p* = 0.0181 and *p* = 0.0133, respectively; Figure 2b).

### 3.3. Prognostic Value of PDK Expression in GC

**Figure 3:**
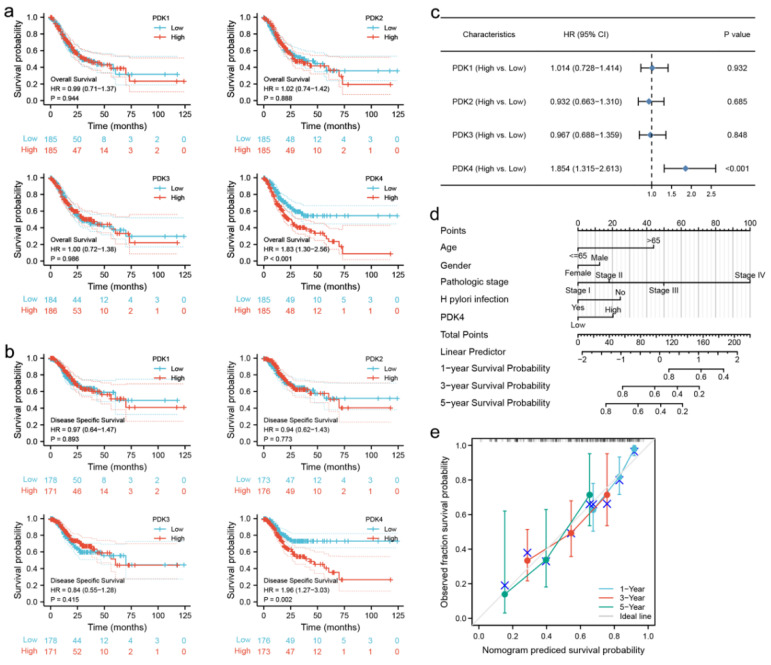
The prognostic value of differently expressed PDK family in GC patients. (**a**) The OS curve of PDK family in GC. (**b**) The DSS curve of PDK family in GC. (**c**) Forest plot of PDKs expression showing PDK4 an independent prognostic factor among the family. (**d**) Nomogram that integrates PDK4 expression and clinicopathological characteristics for predicting the probability of 1-, 3-, & 5-year OS for patients with GC. (**e**) The calibration curve of the nomogram. CI, confidence interval; DSS, disease-specific survival.

To evaluate the potential association between the PDK family and the prognosis of GC patients, we assessed the clinical outcome based on differentially expressed PDKs. According to the survival curves, GC patients with a low transcriptional level of PDK4 were significantly associated with longer OS and DSS (*p* < 0.001 and *p* = 0.002, respectively; Figure 3a,b). In Cox regression analysis (Figure 3c) of all members of the PDK family, PDK4 expression was confirmed to be an independent prognostic factor for predicting clinical outcomes of GC patients (hazard ratio (HR) = 1.854; 95% confidence interval = 1.315–2.613, *p* < 0.001).

On the nomogram, a lower total number of points represented a better prognosis in GC patients (Figure 3d). For example, a 50-year-old (0 points) female (0 points) stage III GC (50 points) patient with Helicobacter pylori infection (0 points) and high PDK4 expression (20 points) received a total score of 70 points. The probabilities of one-, three-, and five- year OS of this patient were approximately >80%, 72%, and 62%, respectively. The calibration curve of the nomogram indicated good prediction efficiency (Figure 3e).

### 3.4. Identification of DEGs of PDK4

**Figure 4:**
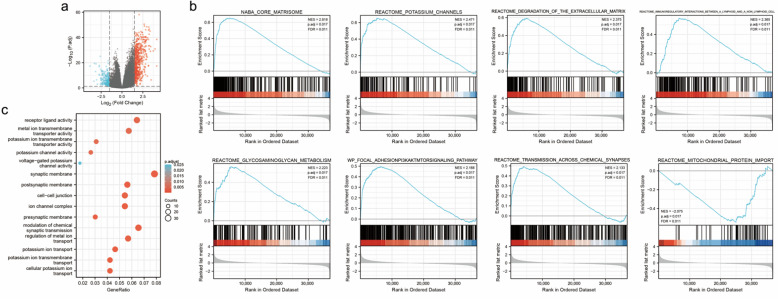
Identification of DEGs and enrichment analyses of PDK4 in GC. (**a**) Volcano plots of the DEGs. (**b**) Bubble chart of GO results displaying DEGs in low- and high- PDK4 expression samples. (**c**) GSEA analysis of the DEGs between low- and high- NOD1 expression groups. DEGs, differentially expressed genes. GO, gene ontology. GSEA, gene set enrichment analysis.

We compared 187 PDK4-low-expression samples with 188 PDK4-high-expression samples of GC from TCGA (Table 1) and identified a total of 774 DEGs, with 546 upregulated genes and 228 downregulated genes (Figure 4a).

### 3.5. Enrichment Analyses of PDK4 in GC

Based on the GO analysis, five of the most significant GO terms, including receptor–ligand activity, synaptic membrane, regulation of metal ion transport, etc. from each enriched biological process (BP), cellular composition (CC), and molecular functions (MFs), that were involved in the regulation of PDK4 interactive genes are presented in the bubble chart (Figure 4b; Table S1).

Furthermore, GSEA identified several PDK4-related pathways and biological processes that are differentially enriched in GC (Figure 4c; Table S2). The results showed that the PDK4 high-expression group was positively enriched (*p* = 0.017 for all) in the core matrisome (NES 2.818), potassium channels (NES 2.471), glycosaminoglycan metabolism (NES 2.223), focal adhesion: PI3K-Akt-mTOR-signaling pathway (NES 2.168), and transmission across chemical synapses (NES 2.133). In addition, the PDK4 low-expression group revealed a significant negative association with the mitochondrial protein import (NES −2.075, *p* = 0.017).

### 3.6. PPI Network and Module Analysis

**Figure 5:**
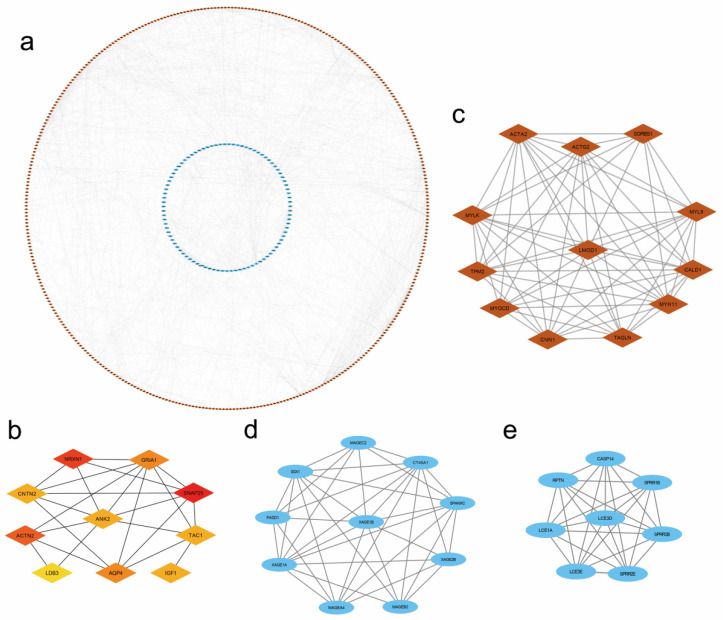
PPI network of PDK4 in GC. (**a**) PPI network constructed using the STRING database. (**b**) The 10 hub genes displayed from red (high degree value) to yellow (low degree value). (**c**) Cluster 1 containing 12 gene nodes and 62 edges, MCODE score = 11.273. (**d**) Cluster 2 containing 10 downregulated nodes and 37 edges, MCODE score = 8.222. (**e**) Cluster 3 containing eight nodes and 28 edges, MCODE score = 8. Red and blue represented Up- and down-regulated genes, respectively. PPI, protein–protein interaction. MCODE: molecular complex detection.

To obtain the interactions between the 774 DEGs of PDK4, a PPI network constructed with 460 nodes and 1838 edges (interaction score >0.4) was identified by the STRING database (Figure 5a). The top 10 hub genes were *SNAP25*, *NRXN1*, *ACTN2*, *GRIA1*, *AQP4*, *CNTN2*, *IGF1*, *TAC1*, *ANK2*, and *LDB3* (Figure 5b, Table S3). Furthermore, three significant gene clusters with scores ≥8.0 were presented (Figure 5c–e). Detailed information on hub genes is presented in Table S3.

### 3.7. Immune Infiltration Analysis of PDK4 Expression in GC

**Figure 6:**
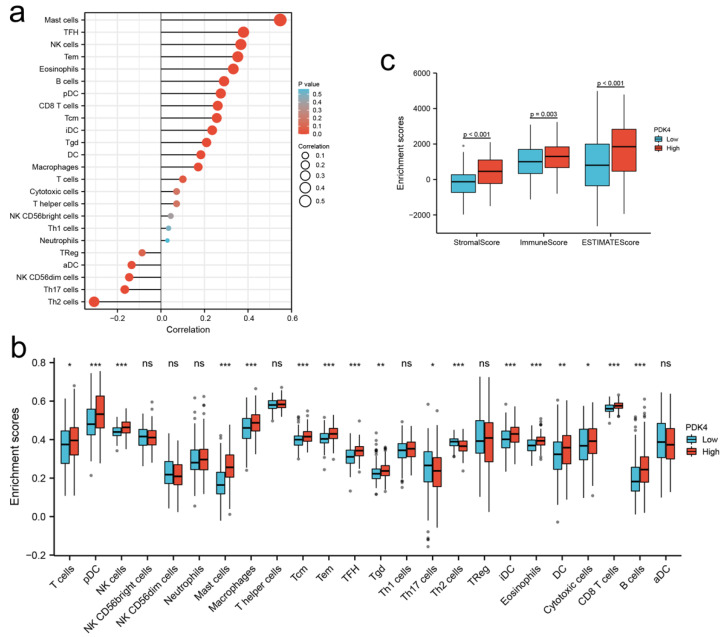
Immune infiltration analysis of PDK4 in GC patients. (**a**) Correlation analysis between PDK4 and related immune cells in GC. (**b**) Comparison of immune infiltration in GC between high- and low-PDK4 expression groups. (**c**) Distribution of stromal score, immune score, and ESTIMATE score between high- and low-PDK4 expression groups in TCGA. TCGA, The Cancer Genome Atlas. * *p* < 0.05, ** *p* < 0.01, *** *p* < 0.001. NK cell, natural killer cell; DC, dendritic cell; pDC, plasmacytoid DC; aDC, activated DC; iDC, interdigitating DC; Tcm, central memory T cell; Tem, effector memory T cell; TFH, follicular helper T cell; Tgd, gammadelta T cell; TReg, regulatory T cell.

Using Spearman’s correlation analysis, we found that PDK4 expression showed a strong positive relationship with mast cell (MCs) infiltration (coefficient 0.548; *p* < 0.001; Figure 6a). Moreover, PDK4 expression positively correlated with the infiltration of other innate immunocytes (e.g., eosinophil, macrophage, dendritic cell [DC], etc.) and acquired immunocytes (e.g., CD8+ T cell, B cell, etc.; Figure 6a, Table S4).

We further compared immune infiltration between high- and low-PDK4 expression groups in GC using the ssGSEA algorithm (Figure 6b, Table S5). In agreement with the results of Spearman’s correlation analysis, the infiltration levels of 15 types of immune cells, including T cell (*p* < 0.05), plasmacytoid DC (pDC; (*p* < 0.001), natural killer (NK) cell (*p* < 0.001), mast cell (MCs; *p* < 0.001), macrophage (*p* < 0.001), central memory T cell (Tcm; *p* < 0.001), effector memory T cell (Tem; *p* < 0.001), follicular helper T cell (TFH; *p* < 0.001), gamma-delta T cell (Tgd; *p* < 0.01), immature DC (iDC; *p* < 0.001), eosinophils (*p* < 0.001), DCs (*p* < 0.01), cytotoxic cell (*p* < 0.05), CD8+ T cell (*p* < 0.001), and B cell (*p* < 0.001) were more significantly upregulated in the PDK4 high-expression group than in the PDK4 low-expression group. In addition, the infiltration of Th17 cell (*p* < 0.05) and Th2 cell (*p* < 0.001) were significantly lower in the PDK4 high-expression group than in the PDK4 low-expression group.

The ESTIMATE algorithm showed that the stromal, immune, and ESTIMATE scores were higher in the PDK4 high-expression group than in the PDK4 low-expression group (*p* < 0.001 for all; Figure 6c).

### 3.8. PDK4 Expression Is Downregulated in GC Tissues

**Figure 7:**
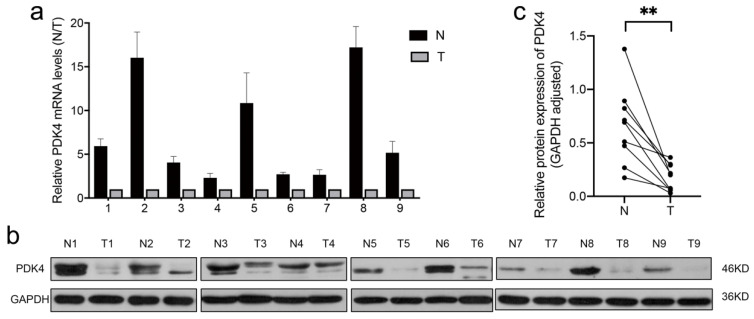
PDK4 expression in GC tissue samples. (**a**) Quantitative real-time PCR analysis of PDK4 expression in 18 pairs of GC tissues and corresponding normal gastric tissues. (**b**,**c**) Results of WB showing that PDK4 expression was significantly downregulated in GC tissues. Data are displayed as means ± SD. ** *p* < 0.01. T, tumor tissues; N, adjacent normal tissues.

To evaluate the pattern of PDK4 expression in GC, we measured PDK4 expression in nine pairs of GC and adjacent normal tissue specimens using real-time PCR and WB. As shown in Figure 7a–c, our data were in line with the results of the database—that is, PDK4 expression was significantly downregulated in GC tissues compared to non-tumor tissues.

### 3.9. High PDK4 Expression in GC Cells and Validation of the Intervention

**Figure 8:**
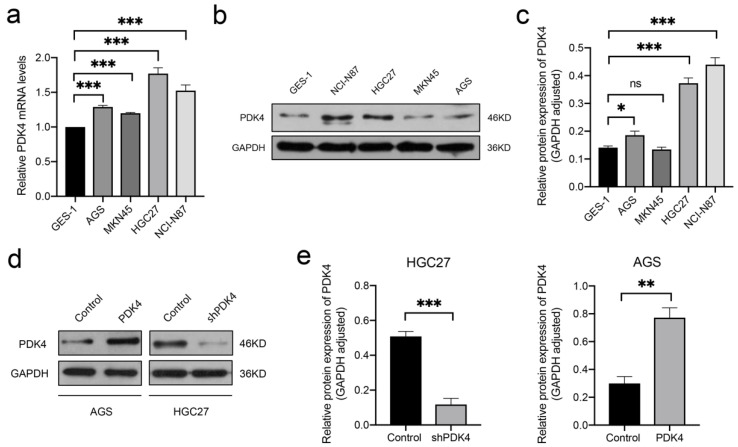
Expression levels of PDK4 in GC cell lines. (**a**–**c**) Results of PCR and WB assays showing higher PDK expression observed in GC cell lines compared to that in GES-1. (**d**,**e**) Results of WB assay showing that relative protein expression of PDK4 was greatly decreased in the shPDK4 groups compared to the control groups in HGC27 and expression of PDK4 was greatly increased in the PDK4 groups compared to the control groups in AGS. Data are displayed as means ± SD. * *p* < 0.05, ** *p* < 0.01, *** *p* < 0.001.

To confirm the results, we also quantified PDK4 expression levels in the normal human gastric epithelial cell line GES-1 and in four GC cells lines (HGC27, NCI-N87, MKN45, and AGS). The results revealed that the mRNA and protein expressions of PDK4 were obviously elevated in all cancer cell lines compared with that in the GES-1 cell line (Figure 8a–c).

According to the results of the RT-qPCR and WB assays, we selected HGC27 to establish a stable PDK4-silenced cell line because of the high level of endogenous PDK4 expression, and used AGS cells for upregulation experiments due to their relatively low PDK4 expression. The lentiviral transfection efficiency was validated using WB (Figure 8d,e). The expression of PDK4 was significantly decreased in the shPDK4 groups compared to the control groups in HGC27 (*p* < 0.001) and significantly increased in the PDK4 groups compared to the control groups in AGS (*p* < 0.01).

### 3.10. PDK4 Expression Regulates the Proliferation and Colony Formation of GC Cells

**Figure 9:**
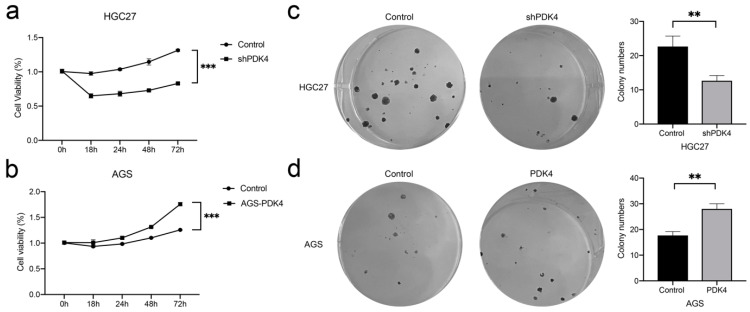
Effect of PDK4 on the tumor cell growth. (**a**,**b**) Growth curves indicating that suppression of PDK4 inhibited HGC27 cell proliferation and overexpression of PDK4 promoted AGS cell proliferation. (**c**,**d**) The tumor colony formation assay indicating that suppression of PDK4 inhibited HGC27 cell proliferation and overexpression of PDK4 promoted AGS cell proliferation. Data are displayed as means ± SD. ** *p* < 0.01, *** *p* < 0.001.

We next investigated the relevance of PDK4 and cell growth using the CCK-8 and colony-formation assays. The analysis of CCK-8 data revealed that, relative to the control group, silencing PDK4 expression notably inhibited GC cell growth (*p* < 0.001, Figure 9a), whereas PDK4 upregulation contributed to enhanced GC cell proliferation (*p* < 0.001, Figure 9b). Similarly, in the colony-formation assay, fewer colonies were observed in the shPDK4 group (*p* < 0.01, Figure 9c), indicating that PDK4 downregulation in HGC27 cells suppressed the colony-forming ability, whereas PDK4 overexpressing cells produced the opposite effect (*p* < 0.05, Figure 9d).

### 3.11. Suppression or Overexpression of PDK4 Could Affect Cell Cycle in GC Cells

**Figure 10:**
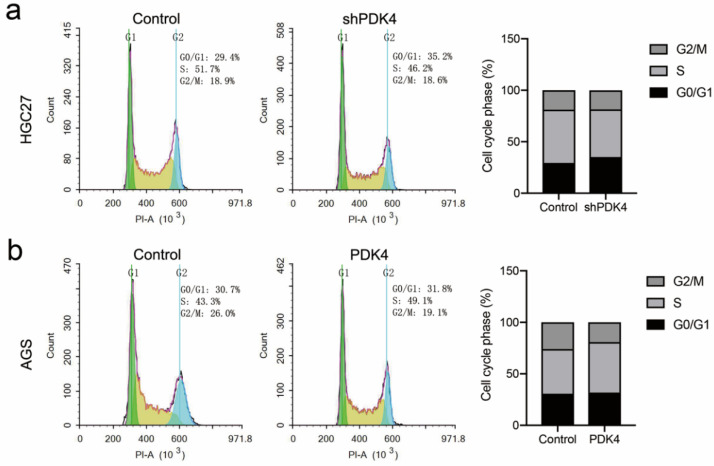
Effect of PDK4 on the cell cycle of HGC27 and AGS cells. (**a**) The flow cytometric detection showing that the S phase populations decreased with G0/G1 cell-cycle arrest in HGC27-shPDK4 cells. (**b**) The percentages of cells showing the S phase populations increased while G2/M phase population decreased in AGS-PDK4 cells.

We performed flow cytometry to determine the effects of PDK4 expression on cell-cycle progression. As shown in Figure 10a, the percentages of shPDK4 cells in the S phase of the cell cycle decreased, whereas the percentage of the cells in the G2/M phase obviously increased. In contrast, PDK4 overexpression in AGS cells drove the cell cycle into S phase and reduced the cell population in the G0/G1 phase compared with the control (Figure 10b). Taken together, PDK4 overexpression induced the S-phase transition, whereas PDK4 downregulation is responsible for G0/G1 cell-cycle arrest.

### 3.12. PDK4 Expression Exerted an Effect on GC Invasion and Migration

**Figure 11:**
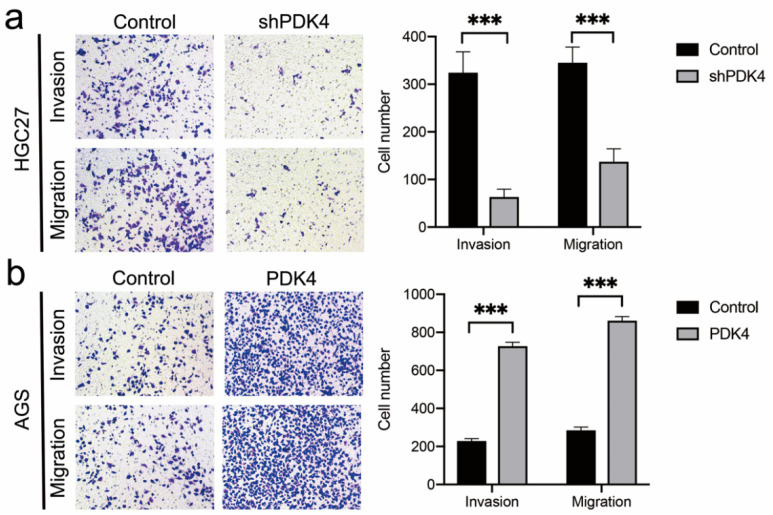
Effect of PDK4 on the invasion and migration of HGC27 and AGS cells. (**a**) The Transwell assay showing that downregulation of PDK4 significantly inhibited the invasion and migration of HGC27 cells. (**b**) Results of Transwell assay showing that the migration and invasion abilities of AGS cells were notably increased after upregulation of PDK4. The bar shows the means ± SD. *** *p* < 0.001.

**Figure 12:**
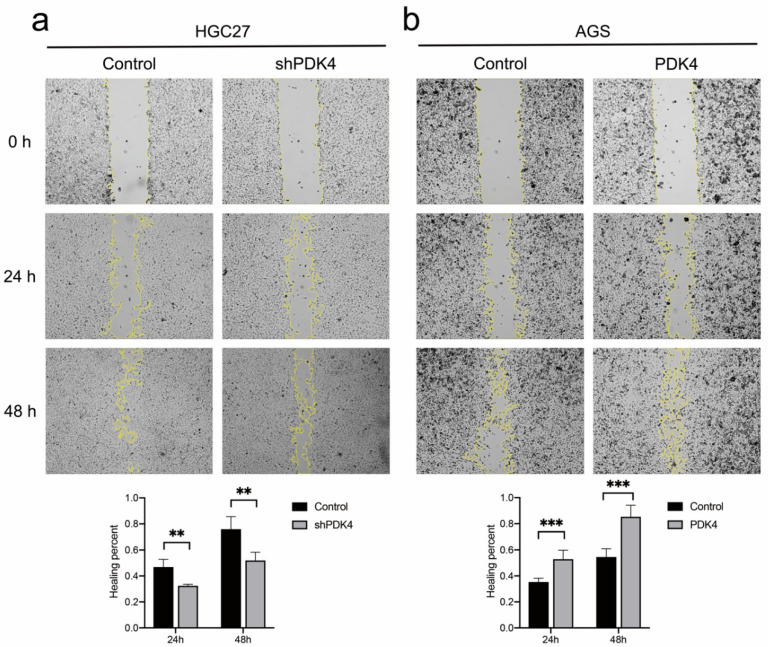
Effect of PDK4 on the migration of HGC27 and AGS cells. (**a**) Wound healing courses showing that migration rates in the shPDK4 groups decreased after both 24 and 48 h for HGC27 cells. (**b**) Wound healing courses showing that migration rates in the PDK4 groups increased after both 24 and 48 h for AGS cells. Data are displayed as means ± SD. ** *p* < 0.01, *** *p* < 0.001.

To probe the influence of PDK4 expression on GC cell invasion and migration abilities, Transwell and scratch wound healing assays were conducted. Our data revealed that the number of cells that passed through the PVDF membrane in the shPDK4-transfected HGC27 cells was less than that in the control group (*p* < 0.001, Figure 11a), whereas upregulation of PDK4 induced more passing cells compared with control cells (*p* < 0.001, Figure 11b). Figure 12a shows that migration rates in the HGC27-shPDK4 groups were lower at both 24 and 48 h compared to the control groups in the cell scratch test (*p* < 0.01). Conversely, we detected higher migration rates in AGS cells with PDK4 overexpression (*p* < 0.001, Figure 12b). These results suggested that knockdown of PDK4 attenuated invasive and migratory abilities of GC cells, whereas upregulation of PDK4 promoted invasion and migration compared with control cells.

## 4. Discussion

GC is one of the most prevalent and deadly cancers worldwide, and is characterized by high malignancy potential and the ease of metastasis [1,2]. Although clinical treatment for GC has been developed, difficulty in early diagnosis and poor prognosis of advanced GC hamper favorable outcomes for GC patients. Therefore, exploring prognostic biomarkers and therapeutic targets of GC may provide novel tools to control the progression of GC.

Tumor cells are characterized by altered energy metabolism, accompanied by other hallmarks of cancer, including immune destruction evasion, proliferative signaling sustentation, cell death resistance, etc. [5]. The phenomenon of the typical metabolic switch from oxidative phosphorylation (OXPHOS) to glycolysis or lactate fermentation and gain of survival potency in hypoxic microenvironments is known as the “Warburg Effect,” under which the acidic conditions make the extracellular matrix extremely unstable and tumor cell metastasis is upregulated. The PDK family, located in the mitochondrial matrix, enhances aerobic glycolysis by switching pyruvate metabolism from the mitochondria to the cytoplasm [7]. Consistently, PDK has been reported to be associated with tumor proliferation, aggressiveness, and therapy resistance in multiple cancers [15]. Among them, PDK4 is the most widely distributed PDK isoform whose upregulation serves an oncogenic role in human colon, bladder, breast, and ovarian cancers [11,12,13,14]. Although the role of PDK1 in the malignancy potential of GC has been identified, PDK4 has not been comprehensively investigated [16]. In this study, we comprehensively analyzed publicly available data, which were verified by molecular biology experiments, and established a prognostic model of PDK4 for GC.

In the present study, we found that the expression of the PDK family was aberrant in multiple cancers. Public data showed that PDK4 was downregulated in GC tissues compared with normal gastric tissues, whereas PDK1/2/3 were upregulated in GC tissues. In line with this, we verified that PDK4 was downregulated in GC tissues using qPCR and WB. In addition, genomic and survival analysis showed that PDK alterations (the most common genetic alterations were “amplification” and “high mRNA”) correlated with poor OS and DFS. Furthermore, GC patients with high PDK4 expression had worse survival than patients with low PDK4 expression. Subsequently, we not only identified that PDK4 was an independent prognostic factor for GC patients, but also proved that the prognostic model constituted with PDK4 expression data and other clinicopathological parameters had a favorable predictive ability with regard to outcomes of GC patients. The abovementioned findings indicated an oncogenic role of PDK4 in GC, which is consistent with our results from qPCR and WB, which showed that PDK4 expression was upregulated in GC cells. This inverse finding of the expression levels of PDK4 between GC cells and GC tissues is discussed further.

The GO analysis indicated that DEGs which are potentially involved in GC were mainly enriched in the regulation of cell adhesion, metal-ion transport, and synaptic activity, which are all significant players in tumor proliferation, invasion, and metastasis [5,17]. Similarly, the enriched GSEA pathways of the high-PDK4 GC group were related to upregulated synaptic activity, enhanced potassium channels, dysfunction of metabolism and mitochondria, upregulation of the core extracellular matrix, focal adhesion, and immunoregulatory interactions. Since the observation made by Otto Warburg with regard to aerobic glycolysis in tumors, the unusual pattern of metabolism and mitochondrial energy supply have been theorized as the underlying basis for oncogenesis in multiple cancers, including GC [18,19,20]. Accordingly, PDK4, which inhibits pyruvate entry into the TCA cycle by inhibiting pyruvate dehydrogenase activity, serves as an important regulator of glucose metabolism and mitochondrial respiration [21]. Interestingly, in a recent study, local vagotomy was unveiled as reversing the metabolic reprogramming of GC at both transcriptomic and metabolic levels [22]. In line with this finding, activation of acetylcholine receptors promotes GC development and progression [17]. Crosstalk between tumor cells and nerves, in turn, induces active neurogenesis, resulting in increased neuronal density and cancer progression. Furthermore, increased neuronal density enhances interactions between neoplastic and perineoplastic tissues (e.g., fibroblasts, immune cells, extracellular matrix, etc.), which induces a microenvironment suitable for tumor progression [22,23,24]. It has been demonstrated that the gastric tumor stage correlated with neural density and activated Wnt signaling, whereas denervation and decreased cholinergic signaling in the GC model suppressed gastric tumorigenesis [24]. In addition, potassium channels, the largest group of ion channels in the human genome, were expressed early during GC progression and were strongly regulated by the tumor hypoxia microenvironment [25]. The upregulation of potassium channels significantly increased Akt activity and vascular endothelial growth factor-A (VEGF-A) secretion in human GC, wherein inhibition of potassium channels can reverse the effect and decreases the expression of hypoxia-inducible factor (HIF)-1α promising anti-angiogenic target [26,27]. Consistently, PDK4 upregulation in suspended untransformed mammary cells conferred anoikis resistance and promoted metastasis via the Warburg effect [28]. Next, our PPI network identified *SNAP25* as the hub gene with the highest degree. According to Rabben et al., who demonstrated that local injection of botulinum toxin A (BoNT-A; synaptosomal nerve-associated protein 25 [SNAP25] inhibitor) presented similar GC suppression as local vagotomy in a recent study, an intratumoral BoNT-A injection enhanced the antitumoral efficacy of systemic RAD001 (also known as everolimus) and CPI-613 (also known as devimistat) [22]. In addition, we have verified that PDK4 upregulation enhanced the ability of GC cells to proliferate, migrate, and invade. Thus, PDK4-targeted therapies may constitute a strategy to hinder cell survival in the tumor microenvironment and protect GC patients against tumor progression and metastasis.

It is well known that the proinflammatory state, which is associated with the recruitment of various immune cells in the tumor microenvironment, acts as an initial step in the oncogenesis of GC and contributes to cancer progression and metastasis [29]. Widely distributed MCs promote tumor development (such as cell proliferation, angiogenesis, invasiveness, metastasis, and survival) in certain neoplasias (e.g., gastric, prostate, and thyroid cancer) [30,31]. It could express inflammatory phenotypes, which would enable the cancer stem cells to develop [32]. MCs exert a pro-tumorigenic role in gastric cancer through the release of angiogenic (VEGF-A, CXCL8, MMP-9) and lymphangiogenic factors (VEGF-C and VEGF-F) [33]. Early MCs support tumor invasiveness by releasing a broad range of matrix metalloproteinases (MMPs) [34]. Moreover, tumor-associated MCs have the potential to shape the tumor microenvironment by establishing crosstalk with other tumor-infiltrating cells [35]. Tumor-specific T helper (Th) cells play a central role in the immune response against cancer. There exist distinct Th cell subsets with different properties. Evidence indicated that Th2 cells promote tumor growth and metastasis, and they are independent protective factors for prostate cancer recurrence after radical prostatectomy [36,37]. Th17 cells drive antitumor immune responses by recruiting immune cells into tumors, activating effector CD8(+) T cells, or even directly by converting toward the Th1 phenotype and producing IFN-γ, which mediates cancer regression in immunotherapy, including adoptive transfer and checkpoint blockade therapy [38]. Consistently, we found that PDK4 overexpression correlated with the increased infiltration of mast cells and the decreased infiltration of Th2 and Th17 cells.

Furthermore, stromal and immune cells, which negatively contribute to tumor purity and prognosis, are considered responsible for angiogenesis and tumor invasion in GC [39,40]. Our ESTIMATE algorithm revealed that PDK4 expression positively correlated with stromal, immune and ESTIMATE scores of GC, which means tumor purity was low in GC samples with high PDK4 expression. This result is also consistent with the above-mentioned survival trend of PDK4, which suggested the oncogenetic nature of PDK4. Furthermore, tumor purity may explain why PDK4 was upregulated in GC cells and downregulated in GC tissues. It has been illustrated that glycolysis plays an indispensable role in development and activation progress of immune cells, including NK cells, T cells, macrophage, B cells, and mast cells. Activated NK cells need glycolysis to produce ATP and effector molecules like IFNγ [41]. A “switch” from OXPHOS to aerobic glycolysis is a hallmark of T cell activation [42]. Th1 and Th17 heavily rely on high glycolytic capacity [43]. Macrophage phenotypes are simply categorized into two types: a pro-inflammatory (M1) and an anti-inflammatory/pro-resolving (M2) profile [44]. In particular, M1 macrophages rely mainly on glycolysis, and M2 cells are more dependent on OXPHOS. Glucose glycolysis promoted early B lymphopoiesis and higher total B lymphocyte numbers [45]. Limited studies that have indicated how mast cells reprogram metabolism remained unclear but most non-IgE mediated activation was based on glycolysis [46]. However, only one article directly evaluated CD4+ T cell populations rely on high glycolytic rates and PDK1 in murine autoimmune encephalomyelitis models [47]. As mentioned previously, most immune cells rely on glycolysis to some extent. However, this requirement in turn restricts their function in the nutrient-limiting tumor microenvironment because tumors impede their access to nutrients and thus impair metabolism and function [43]. Mesenchymal stromal cells (MSCs) possess immune modulating functions. It has been proved that MSCs led to mitigated mTOR signaling, which was accompanied by a weaker glycolytic response toward T-cell activating stimuli [48]. That’s why we supposed that PDK4 expression levels in non-tumor cells are inhibited, which is in contrast with those in tumor cells. In addition, one study using single-cell RNA sequencing revealed that TME consists of a heterogenous cellular milieu and proved that normal epithelial cells were detected in all samples regardless of their origin from tumor, normal or metaplastic tissue. We thought that the lower expression levels of PDK4 in tumor stromal cells and infiltrating immune cells decreased tumor purity with increased PDK4 expression, interfering with the consistency of results in GC cells and in GC tissues. This finding may indicate a complex role of PDK4 in GC tissues.

Nonetheless, the current study has some limitations. First, this study was based on a retrospective analysis. Second, further in vitro experiments should be performed to verify howPDK4 influences tumor progression via cell adhesion, metal ion transport, and synaptic activity in GC. Third, the role that PDK4 plays in immune infiltration status needs to be examined by re-collecting clinical specimens to perform flow cytometry and even single cell sequencing. In vivo experiments are also needed to support our results. The specific role and underlying mechanisms of PDK4 in the development of GC should be validated using further basic experiments and clinical studies.

## 5. Conclusions

This study evaluated and identified the specific oncogenic role and prognostic value of PDK4 in GC. Possible mechanisms mediating the role of PDK4 accrue to the regulatory function in tumor metabolism and the tumor microenvironment. Moreover, we validated the role of PDK4 in human GC cell lines, and the results were consistent with the conclusions of the analysis of data from the public databases. Given the current limited efficacy of GC management strategies, our findings provided an impetus for further research into the pathophysiological mechanisms and the development of efficient therapeutic targets of GC.

## Figures and Tables

**Table 1:** Characteristics of PDK4-high and -low GC samples from TCGA.

Characteristic	Low Expression of PDK4	High Expression of PDK4	*p* Value
n	187	188	
Age, n (%)			0.232
≤65	76 (20.5%)	88 (23.7%)	
>65	110 (29.6%)	97 (26.1%)	
Gender, n (%)			0.352
Female	62 (16.5%)	72 (19.2%)	
Male	125 (33.3%)	116 (30.9%)	
H pylori infection, n (%)			1.000
No	80 (49.1%)	65 (39.9%)	
Yes	10 (6.1%)	8 (4.9%)	
T stage, n (%)			0.211
T1	12 (3.3%)	7 (1.9%)	
T2	41 (11.2%)	39 (10.6%)	
T3	89 (24.3%)	79 (21.5%)	
T4	42 (11.4%)	58 (15.8%)	
N stage, n (%)			0.056
N0	63 (17.6%)	48 (13.4%)	
N1	49 (13.7%)	48 (13.4%)	
N2	39 (10.9%)	36 (10.1%)	
N3	27 (7.6%)	47 (13.2%)	
M stage, n (%)			0.605
M0	170 (47.9%)	160 (45.1%)	
M1	11 (3.1%)	14 (3.9%)	
Pathologic stage, n (%)			0.277
Stage I	30 (8.5%)	23 (6.5%)	
Stage II	63 (17.9%)	48 (13.6%)	
Stage III	70 (19.9%)	80 (22.7%)	
Stage IV	17 (4.8%)	21 (6%)	
OS event, n (%)			<0.001
Alive	131 (34.9%)	97 (25.9%)	
Dead	56 (14.9%)	91 (24.3%)	
DSS event, n (%)			0.003
Alive	145 (41%)	118 (33.3%)	
Dead	33 (9.3%)	58 (16.4%)	

OS, overall survival. DSS, disease-specific survival.

## Data Availability

The datasets generated and/or analyzed during the current study are available in the Cancer Genome Atlas (TCGA; https://www.cancer.gov/about-nci/organization/ccg/research/structural-genomics/tcga (accessed on 10 October 2021)) and the Genotype-Tissue Expression (GTEx; https://commonfund.nih.gov/gtex (accessed on 11 October 2021)).

## Supplementary Materials

The following supporting information can be downloaded at: https://www.mdpi.com/article/10.3390/diagnostics12051101/s1, Table S1: All GO terms with *p* < 0.05; Table S2: All GSEA terms with *p* < 0.05; Table S3: The information of top 10 hub genes; Table S4: Correlation analysis between PDK4 and immune cells in GC; Table S5: Comparison of immune infiltration between high- and low-PDK4 expression groups in GC.
